# Matrix Metalloproteinases Contribute to Neuronal Dysfunction in Animal Models of Drug Dependence, Alzheimer's Disease, and Epilepsy

**DOI:** 10.1155/2011/681385

**Published:** 2011-12-26

**Authors:** Hiroyuki Mizoguchi, Kiyofumi Yamada, Toshitaka Nabeshima

**Affiliations:** ^1^Futuristic Environmental Simulation Center, Research Institute of Environmental Medicine, Nagoya University, Nagoya 464-8601, Japan; ^2^Department of Neuropsychopharmacology and Hospital Pharmacy, Nagoya University Graduate School of Medicine, Nagoya 466-8560, Japan; ^3^Department of Chemical Pharmacology, Meijo University Graduate School of Pharmaceutical Sciences, Nagoya 468-8503, Japan; ^4^Comparative Cognitive Science Institutes, Meijo University, Nagoya 468-8503, Japan

## Abstract

Matrix metalloproteinases (MMPs) and tissue inhibitors of metalloproteinases (TIMPs) remodel the pericellular environment by regulating the cleavage of extracellular matrix proteins, cell surface components, neurotransmitter receptors, and growth factors that mediate cell adhesion, synaptogenesis, synaptic plasticity, and long-term potentiation. Interestingly, increased MMP activity and dysregulation of the balance between MMPs and TIMPs have also been implicated in various pathologic conditions. In this paper, we discuss various animal models that suggest that the activation of the gelatinases MMP-2 and MMP-9 is involved in pathogenesis of drug dependence, Alzheimer's disease, and epilepsy.

## 1. Introduction

Matrix metalloproteinases (MMPs) function to remodel the pericellular environment, primarily by cleaving extracellular matrix (ECM) proteins [1]. This family of enzymes contains more than 20 members, all of which require Zn^2+^ for their enzymatic activities. Several MMPs, including all of the membrane-anchored versions, contain a furin motif (MMP-11, MMP-14, MMP-15, and MMP-16, among others) that mediates intracellular activation of the proteinase prior to its appearance outside of the cell. The activation of the other MMPs requires extracellular proteolytic processing of the secreted zymogens into the active enzymes, a process performed by MMPs or specific proteinases [2, 3].

MMP activity is regulated by interactions with tissue inhibitors of metalloproteinases (TIMPs), a family of multifunctional secreted proteins (TIMP-1–4) that promote growth and regulate the cell cycle in various cell types [1, 4]. Regional and cell-specific expression of MMPs and TIMPs has been detected at various stages of cerebellar development, which may be related to granular cell migration, arborization of Purkinje cells, and synaptogenesis [5]. The regulation of MMPs expression and activity appears to be a very complex process [6]. The expression is regulated at the level of transcription by a variety of growth factors, cytokines, and chemokines although posttranscriptional and epigenetic modification may also contribute.

In an exemplary case of MMP-9, there are a number of gene regulatory sequences driving the MMP-9 expression, with AP1 and NF-*κ*B being the most prominent. MMP-9 is produced by cells in an inactive form. Fully active protein is produced by disruption of the cysteine-zinc interaction and enzymatic removal of the propeptide. The activation of proenzyme is controlled by a cascade of steps involving other MMP and the plasmin system. Pro-MMP-9 can form a complex with TIMP-1, which involves interaction of the C-terminal (noninhibitory) domain of pro-MMP-9 and the C-terminal (noninhibitory) domain of TIMP-1. Moreover, low-density receptor-related protein can act as a receptor for MMP-9, which mediates internalization and degradation of the enzyme.

Of note, extensive cellular migration and remodeling of the ECM are necessary for neural development [7]. It was recently demonstrated that neuronal TIMP-1 [8, 9] and MMP-9 [10] are regulated by synaptic activity, suggesting that the balance between MMPs and TIMPs contributes to activity-dependent reorganization of the neuronal architecture and synaptic physiology. Additionally, MMPs and TIMPs modulate pathophysiologic functional and structural remodeling of the cellular architecture, primarily by regulating the cleavage of ECM proteins, the bioavailability of growth factors and cytokines, and shedding of membrane receptors [1, 3]. Indeed, MMPs have been linked to various pathologic conditions of the central nervous system (CNS), including ischemia, Alzheimer's disease, multiple sclerosis, Parkinson's disease, and malignant glioma. In particular, altered regulation of MMP-2 and MMP-9 has been linked to several nervous system disorders [1]. Therefore, studies of MMPs as key enzymes in both normal and abnormal brain functioning represent a rapidly emerging field.

Keeping these background in mind, we have been investigating the physiologic and pathophysiologic roles for MMP/TIMP in learning and memory, drug dependence, Alzheimer's disease, and epilepsy. In this paper, we discuss potential roles of MMPs—especially MMP-2 and MMP-9—in physiologic and pathophysiologic neural processes.

## 2. MMPs in Emotion and Memory

MMP-9 expression is upregulated, and the protein becomes proteolytically active during the maintenance phase of long-term potentiation (LTP) at CA3–CA1 synapses in the hippocampus [11]. Conversely, TIMP-1 abolishes MMP-9-dependent long-lasting LTP in the prefrontal cortex [12]. These reports suggest a role for MMP-9/TIMP-1 system in neuronal plasticity such as LTP and memory.

FN-439, a broad-spectrum metalloproteinase inhibitor, disrupts reconsolidation of fear memories, which is related to emotionality in a reactivation-dependent manner [13]. Infusing an MMP inhibitor into the dorsal hippocampus disrupted acquisition of spatial memory during the Morris's water maze test [14]. Hippocampal MMP-9 expression transiently increased during memory acquisition in water maze tests, and inhibition of MMP activity with MMP-9 antisense oligonucleotides and an MMP inhibitor altered long-term potentiation and prevented the acquisition of spatial memories during the Morris's water maze test [15]. Conversely, TIMP-1-deficient mice fail to acquire an odor-conditioned learning task, suggesting that the disruption of MMP/TIMP balance disrupt physiologic function such as learning [16]. Interestingly, tissue plasminogen activator and MMPs are also associated with anxiety-like behavior and memory formation, with some studies demonstrating that tissue plasminogen activator in the amygdala promoted stress-induced synaptic plasticity and anxiety-like behavior [17, 18]. Thus, expression of these proteases may be critical for emotion and memory, and deletion of MMPs in mice may induce abnormalities in behavioral tests.

On the other hand, activity-dependent changes in the ECM are thought to alter synaptic architecture and physiology such that the efficiency of synaptic transmission is affected. A recent study identified MMP-9 as a physiologic regulator of N-methyl-D-aspartate (NMDA) receptor-dependent synaptic plasticity and memory. These effects of MMP-9 on NMDA receptors are not mediated by changes in the overall ECM structure or direct cleavage of NMDA receptor subunits, but rather involve an integrin *β*1-dependent pathway [19]. NMDA receptors are important mediators of synaptic plasticity that are central to the neurobiological underpinnings of emotionality, learning, and memory [20]. Thus, MMPs may play critical roles in emotion and memory by extracellularly regulating NMDA receptor signaling.

### 2.1. Emotional and Cognitive Behavioral Phenotypes in MMP-2^(–/–)^ and MMP-9^(–/–)^ Mice

We investigated the roles of MMP-2 and MMP-9 in emotional and cognitive function using mice with targeted deletions of the *MMP-2* or *MMP-9* genes [21]. Emotional changes in MMP-2^(–/–)^ manifested as more frequent rearing in the open-field test and increased time in the open arms and more open arm entries in the elevated plus-arm maze test. Unlike MMP-2^(–/–)^ mice, when MMP-9^(–/–)^ mice were exposed to mildly stressful situations, they showed behavioral responses that indicated reduced anxiety (Table 1). Thus, endogenous MMP-9 may contribute to signaling pathways that modulate emotional behaviors.

MMP-9^(–/–)^ mice also showed impaired long-term object recognition and fear memory, whereas spontaneous alternation behavior in the Y-maze test and short-term memory were normal [21]. MMP-2^(–/–)^ mice do not have any learning or memory phenotypes. These results suggest that MMP-9 plays a role in the formation of long-term memories but not short-term memories (Table 2). Furthermore, Nagy et al. [11] showed that MMP-9 null-mutant mice displayed a significant deficit in long-term hippocampus-dependent memories for context conditioning but not cued conditioning. These results indicate that MMPs play critical roles in synaptic plasticity and hippocampal-dependent memory and that inhibiting MMP activity may compromise the ability of the dorsal hippocampus to reconfigure ECM molecules and interfere with spatial memory acquisition. Although no major defects in brain structures were observed with histochemical examinations, it is possible that neuroadaptation and compensatory mechanisms activated in response to targeted deletions of *MMP-2* and *MMP-9* contributed to the emotional and cognitive phenotypes in the mutant mice.

## 3. MMPs in Drug Dependence

Drug dependence is a complex phenomenon that, in some cases, is mediated by neural plasticity and remodeling of specific brain circuits after repeated exposure to a drug [22, 23]. For example, methamphetamine (METH), a commonly abused drug, causes both acute and long-lasting effects on psychomotor behavior [24–26]; these effects are associated with increased extracellular dopamine levels in the brain, owing to facilitated dopamine release from presynaptic nerve terminals and inhibited dopamine reuptake [27]. The cellular and molecular mechanisms underlying drug dependence are thought to include processes similar to those operating in other forms of synaptic plasticity, such as learning and memory [22, 23, 28]. Nevertheless, although repeated treatment with cocaine or amphetamines alters neural morphology and synaptic connectivity in the mesolimbic system [29, 30], the mechanisms underlying psychostimulant-induced remodeling of synaptic structures have not been determined [31].

### 3.1. METH-Induced Changes in MMP and TIMP Expression in the Brain

We demonstrated that repeated administration of METH led to behavioral sensitization, which was accompanied by the induction of MMP-2, MMP-9, and TIMP-2 expression in various regions of the brain, including the frontal cortex and nucleus accumbens (NAc; Table 3) [32–34]. Our data also showed that MMP-2 and MMP-9 were expressed in neurons and glial cells. Repeated, but not single, METH doses also induced TIMP-2 mRNA and protein expression in neurons [32]. Previous studies also suggested that the MMP/TIMP system is active in neuronal and glial cells and that the expression levels and cellular localization of these proteins are regulated according to developmental and/or functional status of the brain [11, 35]. For instance, during axonal extension, MMPs are located at growth cone tips, where they mediate attachment and detachment of neurons to the matrix substratum, whereas MMPs mediate process extension in oligodendrocytes, suggesting that the regulation of MMPs is an important component of synaptogenesis [2]. Accordingly, MMP-2 and MMP-9 may contribute to structural and functional alterations in the brain following repeated exposure to METH.

### 3.2. Roles of the MMP/TIMP System in METH-Induced Behavioral Sensitization and Reward

We demonstrated that METH-induced behavioral sensitization and conditioned place preference (CPP)—a measure of the rewarding effect of drugs—were markedly attenuated in MMP-2^(–/–)^ and MMP-9^(–/–)^ mice compared with wild-type mice [33], and doxycycline, inhibitor of MMP-2 and MMP-9, blocked METH-induced behavioral sensitization and CPP (Table 4) [32]. Antisense TIMP-2 oligonucleotides (TIMP-AS) inhibited METH-induced increases in TIMP-2 protein expression and enhanced locomotor sensitization, effects that were associated with potentiation of METH-induced dopamine release in the NAc (Table 4) [33]. Accordingly, a net increase in MMP activity may be responsible, at least in part, for the development of METH-induced behavioral sensitization and structural and functional changes in the brain that underlie METH dependence. Indeed, inhibiting MMPs altered functional and structural correlations of deafferentation-induced sprouting, such as remodeling in the dentate gyrus of the hippocampus [36]. Interestingly, Brown et al. [37] demonstrated that MMPs were involved in creating cocaine-associated contextual memories and that reconsolidation of these memories was disrupted by an MMP inhibitor. Further, the authors showed that an MMP inhibitor impaired the expression of memory in response to a cocaine-associated cue after extinction. In addition, elevated MMP-9 activity in the prefrontal cortex contributed to synaptic remodeling that was important for the reactivation of a cocaine-related memory or, alternatively, the modification of a competing extinction memory during reinstatement [38]. Moreover, MMP-9 is elevated in serum of alcohol abusers [39], alcohol treatment increased MMP-1, -2, and -9 activity, and decreased TIMP-1 and -2 in the brain microvascular endothelial cells [40], and inhibiting MMPs attenuated ethanol self-administration [41]. These reports suggest that MMPs may also have a crucial role in drug-associated memory. Because the neurologic underpinnings of learning and memory are closely tied to the development of aberrant behaviors related to drug abuse and addiction [22, 23, 28], MMP-2 and MMP-9 expression may be critical in the acquisition of METH-induced CPP.

Recently, it was reported that MMP-9 expression does not affect METH-induced neurotoxicity and instead contributes to neural remodeling [42]. Further, Conant et al. [43] demonstrated that METH induced ICAM-5 cleavage in murine hippocampus and striatum, which was accompanied by increased MMP-9 protein levels. The authors also showed that the N-terminal ICAM-5 ectodomain stimulated phosphorylation of cofilin via an integrin *β*1-dependent mechanism that was linked to spine maturation. Although additional studies are necessary, these data implicate MMPs in synaptic plasticity associated with drug dependence.

### 3.3. Roles of the MMP/TIMP System in Dopamine Neurotransmission

Behavioral changes induced by METH are linked to its capacity to elevate extracellular dopamine levels by redistributing dopamine from synaptic vesicles to the cytosol and promoting reverse transport [44, 45]. METH-induced behavioral sensitization is associated with enhanced METH-induced increases in extracellular dopamine levels in the NAc [27, 45]. Compared with wild-type mice, MMP-2^(–/–)^ and MMP-9^(–/–)^ mice showed attenuated METH-induced dopamine release in the NAc, behavioral sensitization, and CPP [33]. Sensitization of METH-induced dopamine release in the NAc was enhanced and reduced by TIMP-AS and inhibitors of MMP-2 and MMP-9, respectively. In contrast, infusion of purified human MMP-2 into the NAc significantly potentiated METH-increased dopamine release [32]. Reduced [^3^H]dopamine uptake into striatal synaptosomes was observed in wild-type mice after repeated METH treatment, whereas these changes were significantly attenuated in MMP-2^(–/–)^ and MMP-9^(–/–)^ mice [33]. Reverse activation and internalization of plasmalemmal dopamine transporter are involved in METH-induced increases in extracellular dopamine levels [44, 46]. These results suggest that MMP-2 and MMP-9 play crucial roles in METH-induced behavioral sensitization and reward by regulating METH-induced dopamine release and uptake via dopamine transporters in the NAc.

The sensitivity of dopamine receptors to endogenous and exogenous ligands is an important factor in physiologic and pathologic dopamine neurotransmission, and reduced signaling via dopamine D2 receptors may be an important neuroadaptation in cocaine addiction [47]. G protein signaling in the frontal cortex may play a crucial pathologic role in cocaine sensitization and drug seeking [48]. We have also shown that dopamine receptor agonist-stimulated [^35^S]GTP*γ*S binding is reduced in the frontal cortex of rats sensitized to METH. TIMP-AS and an MMP-2/MMP-9 inhibitor, respectively, potentiated and attenuated the reduction in dopamine D2 receptor agonist-stimulated [^35^S]GTP*γ*S binding. Repeated METH treatment also reduced dopamine D2 receptor agonist-stimulated [^35^S]GTP*γ*S binding in wild-type mice, whereas these changes were significantly attenuated in MMP-2^(–/–)^ and MMP-9^(–/–)^ mice [32]. These results suggest that MMPs and TIMPs are involved in METH-induced dysregulation of dopamine release and receptor signaling. Because dopamine D2 receptors function in feedback inhibition of dopamine release [49, 50], downregulated signaling may enhance METH-induced increases in extracellular dopamine levels.

## 4. MMPs in Alzheimer's Disease

Alzheimer's disease is characterized by the deterioration of cognitive and mental function. The pathology of Alzheimer's disease involves the formation of extracellular deposits of amyloid beta (A*β*) peptide, leading to neuritic plaques and neurofibrillary tangles in the cortex and hippocampus [51, 52]. The neurotoxicity of A*β* has been clearly demonstrated *in vitro* and *in vivo* and shown to involve oxidative stress, perturbed intracellular calcium homeostasis, and activation of apoptosis [53], and neural uptake of extracellular A*β* [54].

Intrahippocampal injections of A*β*, including A*β*1-40, A*β*1-42, and A*β*25-35, have been shown to induce hippocampal damage, learning and memory deficits [55–57], and impairment of the cholinergic system, which contribute to cognitive deficits associated with aging and neurodegenerative diseases [58–60]. Whereas plaques and amyloid fibrils are thought to resist proteolytic degradation, certain proteases may be involved in endogenous mechanisms that clear plaques. A recent study showed that intracerebroventricular infusion of A*β*1-42 induced learning deficits after 12 weeks in 9-month-old mice but not 2.5-month-old mice, suggesting that A*β* infusion resulted in age-dependent and delayed learning deficits independent of A*β* deposition and inflammation [61].

Interestingly, MMP-9 levels are increased in the brains of patients with Alzheimer's disease (Table 5) [62–65]. MMP-9 is expressed in the cytoplasm of neurons, neurofibrillary tangles, senile plaques, and vascular walls in brain tissue from patients with Alzheimer's disease [65]. Moreover, MMP-9 expression is induced in astrocytes in the presence of A*β* peptide [66]. Although MMP-9 cleaves the A*β* peptide at several sites [62, 67], its role in A*β*-induced cognitive dysfunction and neurotoxicity is unclear.

### 4.1. A*β*-Induced MMP Expression in the Brain

Previous study has revealed that both MMP-2 and MMP-9 are induced by the presence of A*β* [67] and highly expressed and secreted by astrocytes [68, 69]. Accordingly, we investigated whether MMP-2 and MMP-9 activities were induced by intracerebroventricular injections of A*β*25-35 and A*β*1-40 using gel zymography [70]. Intracerebroventricular injection of A*β*25-35, A*β*1-40, or A*β*1-42 transiently and dose-dependently increased hippocampal MMP-9 activity and protein expression, whereas MMP-2 was not affected. The injection of a control peptide, A*β*40-1, had no effect on MMP-9 activity and protein expression in the hippocampus, suggesting that the induction of MMP-9 following intracerebroventricular A*β* injection is not due to nonspecific response to brain damage by the insertion of injection needle. Immunohistochemistry revealed increased expression of MMP-9 in both neurons and glial cells in the hippocampus after A*β* administration [70]. When gelatinase activity was analyzed in the hippocampus following intracerebroventricular injection of A*β*1-40 using *in situ* zymography, markedly increased activity was detected in the hippocampal molecular layer in the A*β*1-40-treated group compared with the A*β*40-1-injected group [70].

Moreover, we found that pretreatment with MK-801 inhibited the A*β*-induced increase in MMP-9 expression, indicating that activation of NMDA receptors is involved in A*β*-induced expression of MMP-9 *in vivo* [70].

### 4.2. MMP Inhibition and Reduced A*β*-Induced Cognitive Impairment and Neurotoxicity

Because MMP-9 has been reported to cleave insoluble A*β in vitro* [67], it was thought that MMP inhibitors may potentiate A*β*-induced cognitive dysfunction and neurotoxicity. Our findings do not support this concern, however. In fact, treatment with GM6001, an MMP inhibitor, ameliorated A*β*-induced impairment of recognition memory, suggesting that transient increases in hippocampal MMP-9 activity are associated with the development of A*β*-induced cognitive deficits. The findings obtained with a pharmacologic inhibitor were supported by results showing that intracerebroventricular injection of A*β*1-40 impaired recognition memory in wild-type mice but not MMP-9^(–/–)^ mice [70]. Therefore, even if MMP-9 degrades A*β* or amyloid plaques, it may nonselectively degrade ECM proteins and neural membranes, leading to neuronal dysfunction and cognitive impairment.

Recent evidence has linked MMPs to various pathologic conditions in the CNS, including ischemia, multiple sclerosis, Parkinson's disease, and malignant glioma. This implies that in addition to degrading ECM proteins, MMPs may regulate cell death. In fact, recent studies have indicated that MMP-9 has direct neurotoxic effects [71]. We demonstrated that MMP inhibitor II, which is reportedly highly selective for MMP-2 and MMP-9 [72], blocks A*β*-induced release of lactate dehydrogenase in primary cultured neurons, indicating that MMP-9 contributes to A*β*-induced neuronal cell death. A*β*-induced neurotoxicity was markedly reduced in primary cultured cortical neurons from MMP-9^(–/–)^ mice compared with neurons from wild-type mice. These results suggest that MMP-9 expression in the hippocampus following A*β*1-40 infusion had a causal role in the resulting neurotoxicity and cognitive impairment. On the basis of our findings, it is speculated that A*β*-induced secondary dysfunction such as MMP activation could result in learning deficits by impairing synaptic function in the hippocampus. Our findings highlight the contribution of neural/glial MMP-9 to A*β*-induced neurotoxicity and cognitive impairment and support the idea that for highly selective MMP-9, inhibitors may reduce deleterious proteolytic activity and neuronal death in Alzheimer's disease.

## 5. MMPs in Epilepsy

Seizures cause brain injury via a number of mechanisms that may contribute to neurologic and cognitive deficits in patients with epilepsy. Although seizures induce neuronal death in some situations, they also produce nonlethal pathophysiologic effects on neuronal structures and functions [73]. Kindling is an experimental epilepsy model in which repeated electrical or chemical stimulation of certain forebrain structures triggers progressively more intense electroencephalographic and behavioral seizure activity [74–76]. Once established, kindling results in a permanent state of seizure susceptibility, which may manifest as spontaneous epileptiform seizures [76, 77].

Serum MMP-9 levels and the ratio of MMP-9 to TIMP-1 are elevated in children with various febrile seizures and convulsive status epilepticus [78]. Moreover, expression levels of MMP-9 mRNA increased in response to neuronal depolarization in the rat hippocampus [79], and MMP-9 mRNA was transported to dendrites and synapses in the hippocampal dentate gyrus of kainic acid-treated rats [80]. Jourquin et al. [71] used organotypic cultures to demonstrate increased release and activity of MMP-9 after stimulation with neurotoxic kainate and reduced neuronal cell death following MMP-9 inhibition. TIMP-1 mRNA is also increased in dentate gyrus following seizures, and elevated TIMP-1 mRNA and protein are measured in the hippocampus with seizure [8]. TIMPs are produced by microglia and astrocytes located in cortex and white matter and may play a role in neural regeneration depending upon the degree of expression and the time since injury [81]. Although MMP-9/TIMP-1 is expressed in response to neural activity in some models of epileptogenesis [82–85], the pathophysiologic and etiologic roles of this metalloproteinase, including potential molecular targets during kindling seizure development, are not clear.

### 5.1. MMPs in Kindled Seizure

Mice administered a single pentylenetetrazole (PTZ) dose (20–40 mg/kg) exhibit ear and facial twitching or, at times, convulsive twitching axially throughout the body. PTZ at a dose of 60 mg/kg induces seizures characterized by tonic convulsion, jumping, and wild running. Repeated administration of PTZ at a dose of 40 mg/kg produces chemical kindling, a phenotype associated with a progressive increase in the seizure score. Repeated PTZ treatment increased MMP-9 expression in the hippocampus for at least 24 h after the final dose (Table 6). On the other hand, hippocampal MMP-9 levels were not affected in mice that showed convulsive seizures in response to a single 60 mg/kg PTZ dose. No changes in hippocampal MMP-2 levels were detected following acute or repeated PTZ treatment [86].

We investigated the role of MMP-9 in PTZ-induced kindled seizure using MMP-9^(–/–)^ mice. No difference in the severity of tonic seizures induced by a single PTZ treatment was observed between wild-type and MMP-9^(–/–)^ mice. Repeated PTZ treatment induced kindled seizure in wild-type mice, whereas PTZ-treated MMP-9^(–/–)^ mice showed a marked delay in kindling development although progression of kindled seizure was eventually observed. These results demonstrate that deletion of *MMP-9* attenuated PTZ-induced kindled seizure [86] and agreed with recent findings showing that the synaptic pool of MMP-9 is a critical determinant of seizure development [83]. We also showed that mossy fiber sprouting induced by repeated PTZ treatment was reduced in MMP-9^(–/–)^ mice compared with wild-type mice, suggesting that increased hippocampal MMP-9 activity is associated with the sprouting of mossy fibers [86].

### 5.2. MMP-9-Mediated Maturation of Brain-Derived Neurotrophic Factor and the Development of PTZ-Induced Kindling

Brain-derived neurotrophic factor (BDNF) has been shown to be a potent morphoregulator that controls axon branching and activity-dependent refinement of synapses [87, 88]. BDNF also plays a role in epileptogenesis and kindled seizure. A recent study showed that high-frequency neuronal activity controlled the ratio of extracellular pro-BDNF to mature BDNF by regulating the secretion of extracellular proteases [89]. Of particular note for this paper, proforms of neurotrophin are cleaved and activated by MMPs [2, 3, 90], and MMP-9 converts pro-BDNF to mature BDNF, resulting in tropomyosin-related kinase B (TrkB) activation [2, 91].

Repeated PTZ treatment increased BDNF mRNA and protein levels in the hippocampi of kindled wild-type mice. MMP-9^(–/–)^ mice showed reduced levels of mature BDNF during the early stage of kindling, which may explain slower kindling development in the mutant animals. During the later kindling stage, however, mature BDNF levels were similar in MMP-9^(–/–)^ and wild-type mice. These results suggest that MMP-9 plays a role in kindling development by converting pro-BDNF to mature BDNF in the hippocampus, whereas other factors mediate BDNF maturation during the later kindling stage. This may explain why the seizure response curves for MMP-9^(–/–)^ and wild-type mice differed more at the early stage than at the later stage of kindling [86].

To prove the assumption described above, the BDNF scavenger TrkB-Fc was microinjected into the right ventricle before each PTZ treatment. The treatment significantly suppressed the development of kindling in wild-type mice, whereas no effect was observed in MMP-9^(–/–)^ mice. On the other hand, bilateral injections of pro-BDNF into the hippocampal dentate gyrus significantly enhanced kindling in wild-type mice but not MMP-9^(–/–)^ mice. These findings suggest that MMP-9 is involved in the progression of behavioral phenotypes in kindled mice owing to conversion of pro-BDNF to mature BDNF in the hippocampus. These results likely reflect a relationship between MMP-9 expression and mature BDNF levels in the development of PTZ-induced kindling [86].

Extracellular BDNF stimulates TrkB receptors on the hilar segment of the mossy fiber to induce axonal branching, which may create hyperexcitable dentate circuits [92]. Thus, PTZ-induced kindling may be aggravated through synaptic transformation, including mossy fiber sprouting induced by prolonged MMP-9 activation and subsequent increase in mature BDNF levels. These findings suggest the contribution of neural/glial MMP-9 to epileptogenesis via its activity on extracellular macromolecules such as BDNF.

## 6. Conclusion

As reviewed in this paper, research has begun to elucidate the roles of MMPs in physiologic processes and acute and chronic CNS disorders. Proteolytic mechanisms regulate various developmental and homeostatic processes, whereas inappropriate proteolysis causes or exacerbates a number of CNS disorders. Our previous studies suggest that MMP-9 plays a role in emotional and cognitive behaviors, which may be related to activity-dependent synaptic plasticity and brain development. Furthermore, MMP-9 expression appears to play a crucial role in METH-induced behavioral sensitization by modulating the function of such plasma membrane proteins as dopamine receptors and transporters. MMP-9 also contributes to A*β*-induced neuronal dysfunction and cognitive impairment by nonspecifically destroying the extracellular matrix and neural membranes. Finally, MMP-9 is involved in the progression of behavioral phenotypes in kindled mice by converting pro-BDNF to mature BDNF in the hippocampus. These results suggest that MMP overexpression is associated with structural and functional changes in the cerebral cortex and mesocorticolimbic system, leading to abnormal behaviors following drug treatment. In addition to contributions to various diseases, MMPs have many physiologic roles in such processes as neurogenesis related to memory formation and emotion. More research is needed to understand the diverse roles of these proteases and their potential as therapeutic targets.

## Figures and Tables

**Table 1:** Summary of behavioral changes related to emotional responses in MMP-2^(−/−)^ and MMP-9^(−/−)^ mice.

Test	Parameters	MMP-9^(–/–)^	MMP-2^(–/–)^
Open-field test	Outer sector crossing	±	±
	Inner sector crossing	±	±
	Time in inner sector	±	±
	Number of rearing	⇑	±
	Number of grooming	±	±
	Start latency	⇓	⇓

Elevated plus-arm maze test	Time in open arm	⇑	±
	Open arm entries	⇑	±
	Time in closed arm	⇓	±
	Closed arm entries	±	±

Behavioral changes in MMP-9^(−/−)^ mice indicate that endogenous MMP-9 play a role in emotional behaviors.

±: no change; ⇑: significant increase versus wild-type mice; ⇓: significant decrease versus wild-type mice. Modified from Mizoguchi et al. [21].

**Table 2:** Summary of impaired learning and memory parameters induced by MMP inhibition.

Test (type of memory)	MMP inhibitor	MMP-9^(−/−)^	MMP-2^(−/−)^
Y-maze test (short-term memory)	N.D.	±	⇓
Novel-object recognition test (short-term object memory)	N.D.	±	±
Novel-object recognition test (long-term object memory)	N.D.	⇓	±
Conditioned fear memory test (long-term fear memory)	⇓	⇓	±
Morris's water maze test (hippocampus-dependent spatial memory)	⇓	N.D.	N.D.

Performance of MMP-9^(−/−)^ mice in various memory test indicates impairments of long-term memory in the mutant mice.

±: no change; ⇓: significant decrease versus control or wild-type mice; N.D.: not determined.

Modified from [13–15, 21].

**Table 3:** Changes in MMP and TIMP expression levels and activities induced by drugs of abuse.

Drug	MMP-2	MMP-9	TIMP-2	MMP-3	Method (expression/activity)	References
Methamphetamine (single treatment)	±	±	±	N.D.	mRNA/activity	[32, 33]
Methamphetamine (repeated treatment)	⇑	⇑	⇑	N.D.	mRNA/protein/activity	[32, 33]

Cocaine	N.D.	⇑	N.D.	±	Activity	[38]

Morphine	⇑	N.D.	⇑	N.D.	Protein/activity	Our unpublished data

Alcohol	N.D.	⇑	N.D.	N.D.	Protein	[39]
	⇑	⇑	⇓	N.D.	Protein/activity	[40]

±: no change; ⇑: significant increase versus control mice; N.D.: not determined.

**Table 4:** Summary of changes in addictive behaviors and dopamine release in response to inhibition of the MMP/TIMP system.

Test	Drug	TIMP-2-AS	Doxycycline, FN-439 (MMP inhibitor)	MMP-2^(−/−)^	MMP-9^(−/−)^	References
Hyperlocomotion (single treatment)	Methamphetamine	±	±	±	±	[32, 33]

Locomotor sensitization (repeated treatment)	Methamphetamine	⇑	⇓	⇓	⇓	[32, 33]

Conditioned place preference (rewarding effect, and drug-associated memory)	Methamphetamine	N.D.	⇓	⇓	⇓	[32, 33]
	Cocaine	N.D.	⇓	N.D.	N.D.	[37]

Self-administration	Alcohol	N.D.	⇓	N.D.	N.D.	[41]

Dopamine release	Methamphetamine	⇑	⇓	⇓	⇓	[32, 33]

±: no change; ⇑: significant increase versus control mice; ⇓: significant decrease versus control mice; N.D.: not determined.

TIMP-2-AS: antisense TIMP-2 oligonucleotides.

**Table 5:** MMP protein expression in the brains of patients with Alzheimer's disease.

MMP	Cell types	References
MMP-2	Blood vessels and white matter glia	[63, 64]
MMP-3	Neuron and occasional plaque	[64]
MMP-9	Neuron, neurofibrillary tangle, plaque, and vessel wall	[62–65]

**Table 6:** Summary of changes in brain MMP expression levels and activities induced by chemical stimulants.

Drug	Dose (mg/kg)	Seizure intensity	MMP-2	MMP-9	Method (expression/activity)	References
Pentylene-tetrazole	40	Ear and facial twitching	±	±	Activity	[86]
	40	Kindled seizure (tonic convulsion)	±	⇑	Protein/activity	[86]
	60	Tonic convulsion	±	±	Activity	[86]
	50	Generalized seizures	N.D.	⇑	mRNA	[79]

Kainic acid	9-10	Tonic convulsion	N.D.	⇑	Protein/activity	[71, 83]
	10	Status epilepticus	N.D.	⇑	mRNA	[80]
	10	Convulsion	⇑	⇑	Activity	[82]

Pilocarpine	325	Status epilepticus	±	⇑	Protein/activity	[84]

Bicuculline	1.5–3.0	Convulsion	⇑	⇑	Activity	[82]

±: no change; ⇑: significant increase versus control mice; N.D.: not determined.

## Abbreviations

A*β*: Amyloid beta
BDNF: Brain-derived neurotrophic factor
CNS: Central nervous system
CPP: Conditioned place preference
ECM: Extracellular matrix
METH: Methamphetamine
MMP: Matrix metalloproteinase
NAc: Nucleus accumbens
NMDA: N-methyl-D-aspartate
TIMP: Tissue inhibitor of metalloproteinase
TrkB: Tropomyosin-related kinase B.

## References

[1] Yong VW, Power C, Forsyth P, Edwards DR (2001). Metalloproteinases in biology and pathology of the nervous system. *Nature Reviews Neuroscience*.

[2] Ethell IM, Ethell DW (2007). Matrix metalloproteinases in brain development and remodeling: synaptic functions and targets. *Journal of Neuroscience Research*.

[3] Sternlicht MD, Werb Z (2001). How matrix metalloproteinases regulate cell behavior. *Annual Review of Cell and Developmental Biology*.

[4] Mannello F, Gazzanelli G (2001). Tissue inhibitors of metalloproteinases and programmed cell death: conundrums, controversies and potential implications. *Apoptosis*.

[5] Vaillant C, Didier-Bazès M, Hutter A, Belin MF, Thomasset N (1999). Spatiotemporal expression patterns of metalloproteinases and their inhibitors in the postnatal developing rat cerebellum. *Journal of Neuroscience*.

[6] Dzwonek J, Rylski M, Kaczmarek L (2004). Matrix metalloproteinases and their endogenous inhibitors in neuronal physiology of the adult brain. *FEBS Letters*.

[7] Wright JW, Reichert JR, Davis CJ, Harding JW (2002). Neural plasticity and the brain renin-angiotensin system. *Neuroscience and Biobehavioral Reviews*.

[8] Rivera S, Tremblay E, Timsit S, Canals O, Ben-Ari Y, Khrestchatisky M (1997). Tissue inhibitor of metalloproteinases-1 (TIMP-1) is differentially induced in neurons and astrocytes after seizures: evidence for developmental, immediate early gene, and lesion response. *Journal of Neuroscience*.

[9] Nedivi E, Hevroni D, Naot D, Israell D, Citri Y (1993). Numerous candidate plasticity-related genes revealed by differential cDNA cloning. *Nature*.

[10] Szklarczyk A, Lapinska J, Rylski M, McKay RDG, Kaczmarek L (2002). Matrix metalloproteinase-9 undergoes expression and activation during dendritic remodeling in adult hippocampus. *Journal of Neuroscience*.

[11] Nagy V, Bozdagi O, Matynia A (2006). Matrix metalloproteinase-9 is required for hippocampal late-phase long-term potentiation and memory. *Journal of Neuroscience*.

[12] Okulski P, Jay TM, Jaworski J (2007). TIMP-1 abolishes MMP-9-dependent long-lasting long-term potentiation in the prefrontal cortex. *Biological Psychiatry*.

[13] Brown TE, Wilson AR, Cocking DL, Sorg BA (2009). Inhibition of matrix metalloproteinase activity disrupts reconsolidation but not consolidation of a fear memory. *Neurobiology of Learning and Memory*.

[14] Wright JW, Brown TE, Harding JW (2007). Inhibition of hippocampal matrix metalloproteinase-3 and -9 disrupts spatial memory. *Neural Plasticity*.

[15] Meighan SE, Meighan PC, Choudhury P (2006). Effects of extracellular matrix-degrading proteases matrix metalloproteinases 3 and 9 on spatial learning and synaptic plasticity. *Journal of Neurochemistry*.

[16] Chaillan FA, Rivera S, Marchetti E (2006). Involvement of tissue inhibition of metalloproteinases-1 in learning and memory in mice. *Behavioural Brain Research*.

[17] Pawlak R, Magarinos AM, Melchor J, McEwen B, Strickland S (2003). Tissue plasminogen activator in the amygdala is critical for stress-induced anxiety-like behavior. *Nature Neuroscience*.

[18] Matys T, Pawlak R, Matys E, Pavlides C, McEwen BS, Strickland S (2004). Tissue plasminogen activator promotes the effects of corticotropin- releasing factor on the amygdala and anxiety-like behavior. *Proceedings of the National Academy of Sciences of the United States of America*.

[19] Michaluk P, Mikasova L, Groc L, Frischknecht R, Choquet D, Kaczmarek L (2009). Matrix metalloproteinase-9 controls NMDA receptor surface diffusion through integrin *β*1 signaling. *Journal of Neuroscience*.

[20] Barkus C, McHugh SB, Sprengel R, Seeburg PH, Rawlins JNP, Bannerman DM (2010). Hippocampal NMDA receptors and anxiety: at the interface between cognition and emotion. *European Journal of Pharmacology*.

[21] Mizoguchi H, Ibi D, Takuma K (2010). Alterations of emotional and cognitive behaviors in matrix metalloproteinase-2 and -9-deficient mice. *Open Behavioral Science Journal*.

[22] Sulzer D (2011). How addictive drugs disrupt presynaptic dopamine neurotransmission. *Neuron*.

[23] Bowers MS, Chen BT, Bonci A (2010). AMPA receptor synaptic plasticity induced by psychostimulants: the past, present, and therapeutic future. *Neuron*.

[24] Grant KM, LeVan TD, Wells SM Methamphetamine-associated psychosis.

[25] Kamei H, Nagai T, Nakano H (2006). Repeated methamphetamine treatment impairs recognition memory through a failure of novelty-induced ERK1/2 activation in the prefrontal cortex of mice. *Biological Psychiatry*.

[26] Mizoguchi H, Takuma K, Fukakusa A (2008). Improvement by minocycline of methamphetamine-induced impairment of recognition memory in mice. *Psychopharmacology*.

[27] Nakajima A, Yamada K, Nagai T (2004). Role of tumor necrosis factor-*α* in methamphetamine-induced drug dependence and neurotoxicity. *Journal of Neuroscience*.

[28] Mizoguchi H, Yamada K, Mizuno M (2004). Regulations of methamphetamine reward by extracellular signal-regulated kinase 1/2/ets-like gene-1 signaling pathway via the activation of dopamine receptors. *Molecular Pharmacology*.

[29] Robinson TE, Kolb B (1997). Persistent structural modifications in nucleus accumbens and prefrontal cortex neurons produced by previous experience with amphetamine. *Journal of Neuroscience*.

[30] Nestler EJ (2001). Molecular basis of long-term plasticity underlying addiction. *Nature Reviews Neuroscience*.

[31] Yamada K, Nabeshima T (2008). Endogenous modulators for drug dependence. *Biological and Pharmaceutical Bulletin*.

[32] Mizoguchi H, Yamada K, Mouri A (2007). Role of matrix metalloproteinase and tissue inhibitor of MMP in methamphetamine-induced behavioral sensitization and reward: implications for dopamine receptor down-regulation and dopamine release. *Journal of Neurochemistry*.

[33] Mizoguchi H, Yamada K, Niwa M (2007). Reduction of methamphetamine-induced sensitization and reward in matrix metalloproteinase-2 and -9-deficient mice. *Journal of Neurochemistry*.

[34] Mizoguchi H, Yamada K, Nabeshima T (2008). Neuropsychotoxicity of abused drugs: involvement of matrix metalloproteinase-2 and -9 and tissue inhibitor of matrix metalloproteinase-2 in methamphetamine-induced behavioral sensitization and reward in rodents. *Journal of Pharmacological Sciences*.

[35] Vaillant C, Didier-Bazès M, Hutter A, Belin MF, Thomasset N (1999). Spatiotemporal expression patterns of metalloproteinases and their inhibitors in the postnatal developing rat cerebellum. *Journal of Neuroscience*.

[36] Reeves TM, Prins ML, Zhu J, Povlishock JT, Phillips LL (2003). Matrix metalloproteinase inhibition alters functional and structural correlates of deafferentation-induced sprouting in the dentate gyrus. *Journal of Neuroscience*.

[37] Brown TE, Forquer MR, Cocking DL, Jansen HT, Harding JW, Sorg BA (2007). Role of matrix metalloproteinases in the acquisition and reconsolidation of cocaine-induced conditioned place preference. *Learning and Memory*.

[38] Brown TE, Forquer MR, Harding JW, Wright JW, Sorg BA (2008). Increase in matrix metalloproteinase-9 levels in the rat medial prefrontal cortex after cocaine reinstatement of conditioned place preference. *Synapse*.

[39] Sillanaukee P, Kalela A, Seppä K, Höyhtyä M, Nikkari ST (2002). Matrix metalloproteinase-9 is elevated in serum of alcohol abusers. *European Journal of Clinical Investigation*.

[40] Haorah J, Schall K, Ramirez SH, Persidsky Y (2008). Activation of protein tyrosine kinases and matrix metalloproteinases causes blood-brain barrier injury: novel mechanism for neurodegeneration associated with alcohol abuse. *GLIA*.

[41] Smith AW, Nealey KA, Wright JW, Walker BM (2011). Plasticity associated with escalated operant ethanol self-administration during acute withdrawal in ethanol-dependent rats requires intact matrix metalloproteinase systems. *Neurobiology of Learning and Memory*.

[42] Liu Y, Brown S, Shaikh J, Fishback JA, Matsumoto RR (2008). Relationship between methamphetamine exposure and matrix metalloproteinase 9 expression. *NeuroReport*.

[43] Conant K, Lonskaya I, Szklarczyk A (2011). Methamphetamine-associated cleavage of the synaptic adhesion molecule intercellular adhesion molecule-5. *Journal of Neurochemistry*.

[44] Sulzer D, Chen TK, Lau YY, Kristensen H, Rayport S, Ewing A (1995). Amphetamine redistributes dopamine from synaptic vesicles to the cytosol and promotes reverse transport. *Journal of Neuroscience*.

[45] Nagai T, Noda Y, Ishikawa K (2005). The role of tissue plasminogen activator in methamphetamine-related reward and sensitization. *Journal of Neurochemistry*.

[46] Khoshbouei H, Sen N, Guptaroy B (2004). N-terminal phosphorylation of the dopamine transporter is required for amphetamine-induced efflux. *PLoS Biology*.

[47] Goldstein RZ, Volkow ND (2002). Drug addiction and its underlying neurobiological basis: neuroimaging evidence for the involvement of the frontal cortex. *American Journal of Psychiatry*.

[48] Bowers MS, McFarland K, Lake RW (2004). Activator of G protein signaling 3: a gatekeeper of cocaine sensitization and drug seeking. *Neuron*.

[49] Bowyer JF, Weiner N (1987). Modulation of the Ca^++^-evoked release of [3H]dopamine from striatal synaptosomes by dopamine (D2) agonists and antagonists. *Journal of Pharmacology and Experimental Therapeutics*.

[50] Lindgren N, Xu ZQD, Herrera-Marschitz M, Haycock J, Hökfelt T, Fisone G (2001). Dopamine D2 receptors regulate tyrosine hydroxylase activity and phosphorylation at Ser40 in rat striatum. *European Journal of Neuroscience*.

[51] Palmer AM (2011). Neuroprotective therapeutics for Alzheimers disease: progress and prospects. *Trends in Pharmacological Sciences*.

[52] Yamada K, Nabeshima T (2000). Animal models of Alzheimer’s disease and evaluation of anti-dementia drugs. *Pharmacology and Therapeutics*.

[53] Takuma K, Yan SS, Stern DM, Yamada K (2005). Mitochondrial dysfunction, endoplasmic reticulum stress, and apoptosis in Alzheimer’s disease. *Journal of Pharmacological Sciences*.

[54] Takuma K, Fang F, Zhang W (2009). RAGE-mediated signaling contributes to intraneuronal transport of amyloid-*β* and neuronal dysfunction. *Proceedings of the National Academy of Sciences of the United States of America*.

[55] Yamada K, Takayanagi M, Kamei H (2005). Effects of memantine and donepezil on amyloid *β*-induced memory impairment in a delayed-matching to position task in rats. *Behavioural Brain Research*.

[56] Wang D, Noda Y, Zhou Y (2007). The allosteric potentiation of nicotinic acetylcholine receptors by galantamine ameliorates the cognitive dysfunction in beta amyloid 25–35 I.c.v.-injected mice: involvement of dopaminergic systems. *Neuropsychopharmacology*.

[57] Alkam T, Nitta A, Mizoguchi H, Itoh A, Nabeshima T (2007). A natural scavenger of peroxynitrites, rosmarinic acid, protects against impairment of memory induced by A*β*25–35. *Behavioural Brain Research*.

[58] Tran MH, Yamada K, Olariu A, Mizuno M, Ren XH, Nabeshima T (2001). Amyloid beta-peptide induces nitric oxide production in rat hippocampus: association with cholinergic dysfunction and amelioration by inducible nitric oxide synthase inhibitors. *The FASEB Journal*.

[59] Yamada K, Tanaka T, Han D, Senzaki K, Kameyama T, Nabeshima T (1999). Protectiye effects of idebenone and *α*-tocopherol on *β*-amyloid-(1-42)-induced learning and memory deficits in rats: implication of oxidative stress in *β*-amyloid-induced neurotoxicity in vivo. *European Journal of Neuroscience*.

[60] Zussy C, Brureau A, Delair B (2011). Time-course and regional analyses of the physiopathological changes induced after cerebral injection of an amyloid *β* fragment in rats. *American Journal of Pathology*.

[61] Malm T, Ort M, Tähtivaara L (2006). *β*-amyloid infusion results in delayed and age-dependent learning deficits without role of inflammation or *β*-amyloid deposits. *Proceedings of the National Academy of Sciences of the United States of America*.

[62] Backstrom JR, Lim GP, Cullen MJ, Tökés ZA (1996). Matrix metalloproteinase-9 (MMP-9) is synthesized in neurons of the human hippocampus and is capable of degrading the amyloid-*β* peptide (1–40). *Journal of Neuroscience*.

[63] Backstrom JR, Miller CA, Tokes ZA (1992). Characterization of neutral proteinases from Alzheimer-affected and control brain specimens: identification of calcium-dependent metalloproteinases from the hippocampus. *Journal of Neurochemistry*.

[64] Baig S, Kehoe PG, Love S (2008). MMP-2, -3 and -9 levels and activity are not related to A*β* load in the frontal cortex in Alzheimer’s disease. *Neuropathology and Applied Neurobiology*.

[65] Asahina M, Yoshiyama Y, Hattori T (2001). Expression of matrix metalloproteinase-9 and urinary-type plasminogen activator in Alzheimer’s disease brain. *Clinical Neuropathology*.

[66] Deb S, Gottschall PE (1996). Increased production of matrix metalloproteinases in enriched astrocyte and mixed hippocampal cultures treated with *β*-amyloid peptides. *Journal of Neurochemistry*.

[67] Yan P, Hu X, Song H (2006). Matrix metalloproteinase-9 degrades amyloid-*β* fibrils in vitro and compact plaques in situ. *Journal of Biological Chemistry*.

[68] Deb S, Zhang JW, Gottschall PE (2003). *β*-amyloid induces the production of active, matrix-degrading proteases in cultured rat astrocytes. *Brain Research*.

[69] Muir EM, Adcock KH, Morgenstern DA (2002). Matrix metalloproteases and their inhibitors are produced by overlapping populations of activated astrocytes. *Molecular Brain Research*.

[70] Mizoguchi H, Takuma K, Fukuzaki E (2009). Matrix metalloprotease-9 inhibition improves amyloid *β*-mediated cognitive impairment and neurotoxicity in mice. *Journal of Pharmacology and Experimental Therapeutics*.

[71] Jourquin J, Tremblay E, Décanis N (2003). Neuronal activity-dependent increase of net matrix metalloproteinase activity is associated with MMP-9 neurotoxicity after kainate. *European Journal of Neuroscience*.

[72] Tamura Y, Watanabe F, Nakatani T (1998). Highly selective and orally active inhibitors of type IV collagenase (MMP-9 and MMP-2): N-sulfonylamino acid derivatives. *Journal of Medicinal Chemistry*.

[73] Zeng LH, Xu L, Rensing NR, Sinatra PM, Rothman SM, Wong M (2007). Kainate seizures cause acute dendritic injury and actin depolymerization in vivo. *Journal of Neuroscience*.

[74] Kokaia M (2011). Seizure-induced neurogenesis in the adult brain. *European Journal of Neuroscience*.

[75] Han D, Yamada K, Senzaki K, Xiong H, Nawa H, Nabeshima T (2000). Involvement of nitric oxide in pentylenetetrazole-induced kindling in rats. *Journal of Neurochemistry*.

[76] Wahab A, Albus K, Gabriel S, Heinemann U (2010). In search of models of pharmacoresistant epilepsy. *Epilepsia*.

[77] Löscher W, Köhling R (2010). Functional, metabolic, and synaptic changes after seizures as potential targets for antiepileptic therapy. *Epilepsy and Behavior*.

[78] Suenaga N, Ichiyama T, Kubota M, Isumi H, Tohyama J, Furukawa S (2008). Roles of matrix metalloproteinase-9 and tissue inhibitors of metalloproteinases 1 in acute encephalopathy following prolonged febrile seizures. *Journal of the Neurological Sciences*.

[79] Rylski M, Amborska R, Zybura K (2009). JunB is a repressor of MMP-9 transcription in depolarized rat brain neurons. *Molecular and Cellular Neuroscience*.

[80] Konopacki FA, Rylski M, Wilczek E (2007). Synaptic localization of seizure-induced matrix metalloproteinase-9 mRNA. *Neuroscience*.

[81] Wright JW, Harding JW (2004). The brain angiotensin system and extracellular matrix molecules in neural plasticity, learning, and memory. *Progress in Neurobiology*.

[82] Zhang JW, Deb S, Gottschall PE (1998). Regional and differential expression of gelatinases in rat brain after systemic kainic acid or bicuculline administration. *European Journal of Neuroscience*.

[83] Wilczynski GM, Konopacki FA, Wilczek E (2008). Important role of matrix metalloproteinase 9 in epileptogenesis. *Journal of Cell Biology*.

[84] Kim GW, Kim HJ, Cho KJ, Kim HW, Cho YJ, Lee BI (2009). The role of MMP-9 in integrin-mediated hippocampal cell death after pilocarpine-induced status epilepticus. *Neurobiology of Disease*.

[85] Takács E, Nyilas R, Szepesi Z (2010). Matrix metalloproteinase-9 activity increased by two different types of epileptic seizures that do not induce neuronal death: a possible role in homeostatic synaptic plasticity. *Neurochemistry International*.

[86] Mizoguchi H, Nakade J, Tachibana M (2011). Matrix metalloproteinase-9 contributes to kindled seizure development in pentylenetetrazole-treated mice by converting pro-BDNF to mature BDNF in the hippocampus. *Journal of Neuroscience*.

[87] Cabelli RJ, Hohn A, Shatz CJ (1995). Inhibition of ocular dominance column formation by infusion of NT-4/5 or BDNF. *Science*.

[88] Horch HW, Krüttgen A, Portbury SD, Katz LC (1999). Destabilization of cortical dendrites and spines by BDNF. *Neuron*.

[89] Nagappan G, Zaitsev E, Senatorov VV, Yang J, Hempstead BL, Lu B (2009). Control of extracellular cleavage of ProBDNF by high frequency neuronal activity. *Proceedings of the National Academy of Sciences of the United States of America*.

[90] Lee R, Kermani P, Teng KK, Hempstead BL (2001). Regulation of cell survival by secreted proneurotrophins. *Science*.

[91] Jung JH, Park MH, Choi SY, Koh JY (2005). Activation of the Trk signaling pathway by extracellular zinc. Role of metalloproteinases. *Journal of Biological Chemistry*.

[92] Koyama R, Yamada MK, Fujisawa S, Katoh-Semba R, Matsuki N, Ikegaya Y (2004). Brain-derived neurotrophic factor induces hyperexcitable reentrant circuits in the dentate gyrus. *Journal of Neuroscience*.

